# Cost-effectiveness of a medication event monitoring system for tuberculosis management in Morocco

**DOI:** 10.1371/journal.pone.0267292

**Published:** 2022-04-19

**Authors:** Jangmi Yang, Hae-Young Kim, Seup Park, Ilham Sentissi, Nathan Green, Byung Kwon Oh, Yujin Kim, Kyung Hyun Oh, Eunseong Paek, Young Joon Park, In-Hwan Oh, Seung Heon Lee

**Affiliations:** 1 National Evidence Based Health Care Collaborating Agency, Seoul, Republic of Korea; 2 Department of Population Health, New York University Grossman School of Medicine, New York, NY, United States of America; 3 Global Care International, Seoul, Republic of Korea; 4 Chief Public Health Service and Epidemiological Surveillance, Moroccan League Against Tuberculosis (Ligue Marocaine de Lute Contre la Tuberculosis, LMCT), Rabat, Morocco; 5 Department of Statistical Science, University College London, London, United Kingdom; 6 Korean Institute of Tuberculosis, Korean National Tuberculosis Association, Cheongju, Republic of Korea; 7 End TB and Leprosy Unit, World Health Organization Regional Office for the Western Pacific, Manila, Philippines; 8 Department of Preventive Medicine, School of Medicine, Kyung Hee University, Seoul, Republic of Korea; 9 Division of Pulmonary, Sleep and Critical Care Medicine, Department of Internal Medicine, Korea University Ansan Hospital, Ansan-City, Republic of Korea; McGill University, CANADA

## Abstract

**Background:**

Digital health technologies have been used to enhance adherence to TB medication, but the cost-effectiveness remains unclear.

**Methods:**

We used the real data from the study conducted from April 2014 to December 2020 in Morocco using a smart pillbox with a web-based medication monitoring system, called Medication Event Monitoring Systems (MEMS). Cost-effectiveness was evaluated using a decision analysis model including Markov model for Multi-drug resistant (MDR) TB from the health system perspective. The primary outcome was the incremental cost-effectiveness ratio (ICER) per disability adjusted life-year (DALY) averted. Two-way sensitive analysis was done for the treatment success rate between MEMS and standard of care.

**Results:**

The average total per-patient health system costs for treating a new TB patient under MEMS versus standard of care were $398.70 and $155.70, respectively. The MEMS strategy would reduce the number of drug-susceptible TB cases by 0.17 and MDR-TB cases by 0.01 per patient over five years. The ICER of MEMS was $434/DALY averted relative to standard of care, and was most susceptible to the TB treatment success rate of both strategies followed by the managing cost of MEMS.

**Conclusion:**

MEMS is considered cost-effective for managing infectious active TB in Morocco.

## Introduction

Tuberculosis (TB) is the leading cause of mortality among infectious diseases, accounting for 1.5 million deaths worldwide in 2018 [1]. In high TB burden countries, the successful completion of TB treatment is one of the critical TB control strategies to prevent TB transmission through the community [2]. Poor adherence to TB treatment can increase the risk of treatment failure, relapse, as well as the development of drug resistance [3]. However, adherence to TB treatment is often suboptimal despite various treatment interventions, including directly observed treatment (DOT).

Recently, digital health technologies and management have been used to enhance adherence to TB medication. Many studies have reported that digital adherence technology (DAT) substantially improved adherence [4] and led to cost savings up to 58% compared to traditional DOT [5]. In 2017, the World Health Organization (WHO) reported that DAT for TB treatment can be used as a substitute of traditional DOTS [6] and recommended its implementation as part of national tuberculosis control programs (NTP) [5]. In order to scale up DAT at a national level, it is important to understand the cost-effectiveness of DAT [7], considering TB burden and the strategic TB program in a given setting [8]. Furthermore, financial and case management strategies should be reassessed to accomplish the goals of global TB strategy [9].

In Morocco, where TB incidence in 2018 was 99/100,000 nationwide and 130/100,000 in cities like Kenitra [10], the overall treatment success rate was 84%, and the loss to follow-up (non-adherence) rate was as high as 15% [11]. We previously reported the demonstrable effectiveness of a smart pillbox (SP) with a web-based medication monitoring system, called a Medication Event Monitoring System (MEMS), as a tailored adherence-monitoring intervention to improve patients’ adherence to TB treatment among active TB patients in Morocco [12]. We used the epidemiological and cost data from this study to evaluate the cost-effectiveness of MEMS to monitor TB treatment among infectious active TB patients in Morocco.

## Methods

### KOICA project in Morocco

The study was conducted in Salé, Morocco by the Korean International Cooperation Agency (KOICA) in collaboration with Global Care International and the Ministry of Health and Welfare of Morocco from April 2014 to December 2020. Briefly, the study enrolled and provided patients a smart pillbox with a web-based medication monitoring system, called MEMS, which reminded patients to take medication at certain times [13]. MEMS as a digital health technology is a medication storage device that sends the data in real-time via a subscriber identity module (SIM) card to a web-based application based on a tele-communication interface (Wi-Fi, 3G/4G, Ethernet). For installation, a staff visited their home and installed the device with the demonstration and explanations about the manual.

MEMS were applied after one to two weeks of routine direct supervision for medication at the treatment site, depending on the circumstances.

The system provided regular statistical monitoring results including drug adherence status according to time, region, and patient information to staffs. When patients failed to take their medication, staffs made phone calls or home visits to patients. This original study compared patients who received a MEMS (n = 206) with patients who received standard TB care (n = 141) among new infectious active TB patients in five rural health centers of Sale area. The results of this study showed that MEMS increased TB treatment success rate and decreased the lost to follow-up rate overall significantly (P<0.001).

### TB guidelines in Morocco

The national TB treatment guidelines of Morocco recommend two months of rifampin, isoniazid, pyrazinamide, and Ethambutol (2RHZE) followed by four months of rifampicin and isoniazid (4RH) for new smear-positive cases [14, 15].

Direct supervision at the treatment site for two months is recommended (modified DOT) before self-administered treatment (SAT). Retreatment regimens include two months of streptomycin, rifampin, isoniazid, pyrazinamide, and Ethambutol (2SRHZE), followed by one month of RHZE and five months of RHE (1RHZE/5RHE). Multi-drug resistant (MDR) TB treatment consists of six months of intensive phase with injectable drugs in addition to levofloxacin, ethionamide, cycloserine, pyrazinamide, and ethambutol, and 18 months of continuation phase without injectable drugs before the WHO TB treatment guidelines were updated in 2018 [15].

### Overview of the Markov model

**Fig 1:**
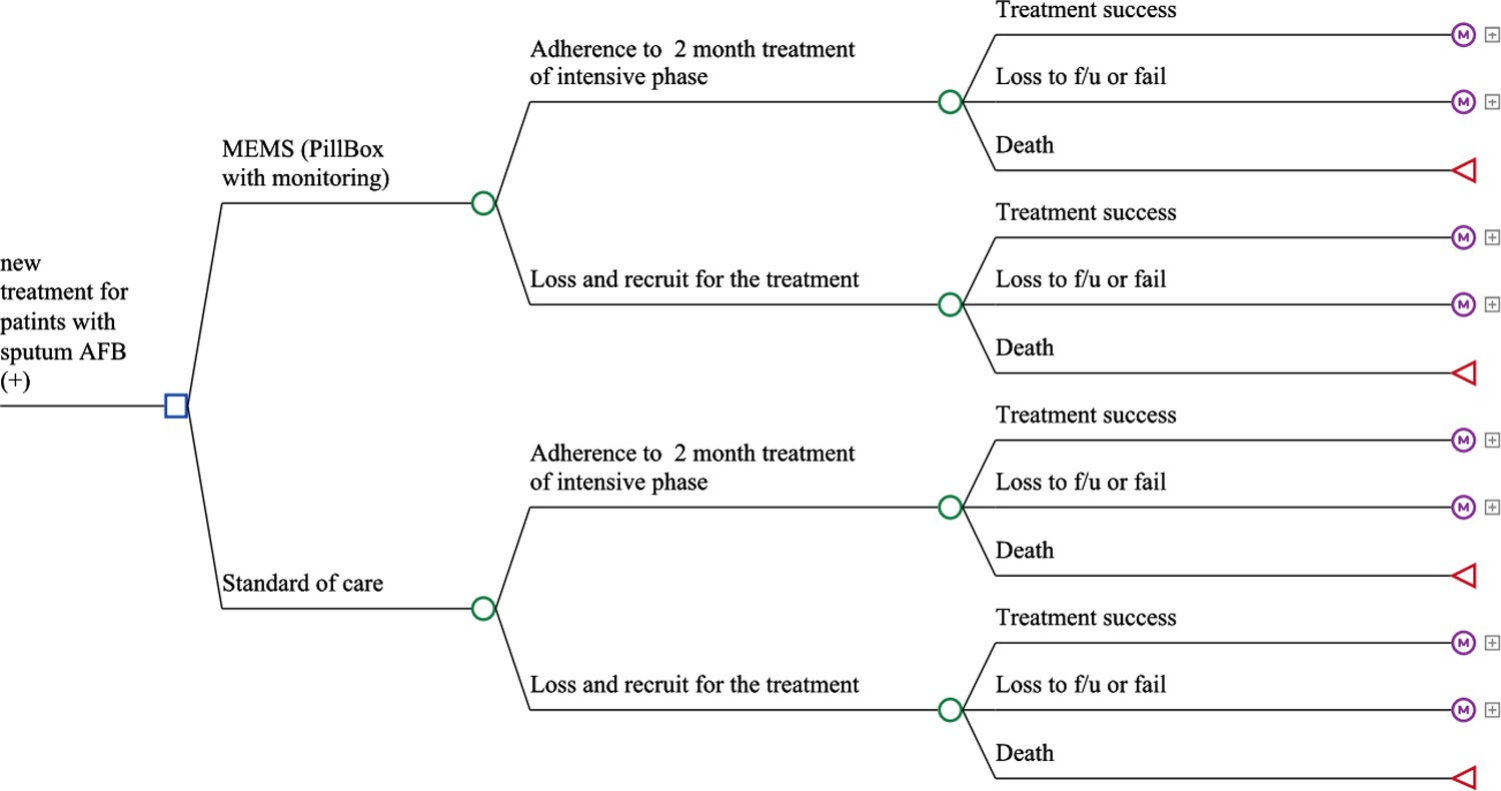
Decision analytic models that represent different Markov health statuses. Diagrams end in Markov nodes (circled M) and terminal nodes (triangles). Markov nodes denote additional subtrees as described in Fig 2. AFB = acid-fast bacilli; MEMS = medication event monitoring system; f/u = follow up.

**Fig 2:**
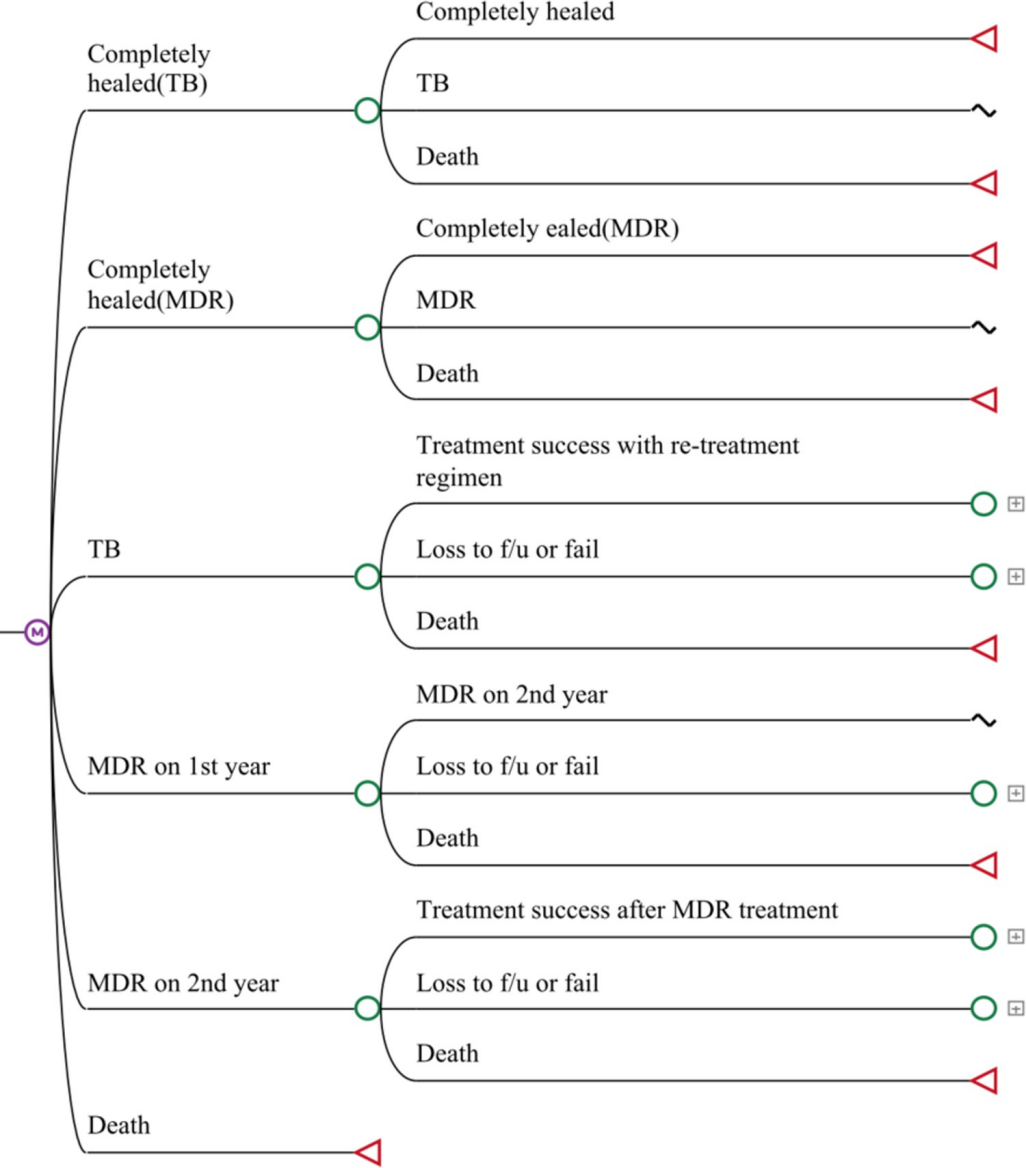
Markov model representing health states and possible transitions between states. Initial probabilities were varied to model the progression of each sub-group through the Markov models. TB = tuberculosis; MDR = multi-drug resistant tuberculosis; f/u = follow up.

We used epidemiology and costing data from the KOICA project to perform a cost-effectiveness analysis from the health system perspective in a hypothetical cohort of infectious TB patients with acid-fast bacilli (AFB) smear-positive results in Morocco. We used a five-health state Markov model to evaluate two strategies for the first line treatment of drug-susceptible TB: 1) standard of care by modified DOT (Standard of Care, SoC); or 2) MEMS. Based on the WHO guideline [16], we included five health states for treatment results as follows: treatment success, loss to follow up or failure, death, complete healing, or recurring TB or MDR-TB (Figs 1 and 2). Based on the treatment outcomes and effectiveness of first-line treatment, patients were categorized into the completion of first-line treatment, recurring TB or MDR-TB, or mortality. Since the first two months of the intensive treatment phase is critical for sputum conversion in sputum AFB smear (+) patients, adherence to the initial two months of treatment was separately categorized for the initial health outcomes.

The patients lost within the initial two months of treatment were assumed to be recruited again for drug-susceptible treatment. We assumed that drug susceptibility testing did not influence treatment because all treatment regimens were considered standardized medications. We limited our analysis to 5-year time horizons to accommodate the following lengths of treatment: six months for initial treatment regimens, eight months for retreatment regimens, and 24 months for MDR-TB treatment regimens with two cycles. All analyses were performed using TreeAge 2019 R2.0 software (Williamstown, MA, USA).

### Epidemiological parameters and cost measurement

Epidemiological parameters were determined from literature review and real data of the KOICA project (Table 1). We conducted a mixed-costing (top-down and bottom-up) for both SoC and MEMS strategies (Table 2). We collected cost and resource-use data related with key activity component (i.e. adherence, management, treatment outcomes, diagnosis with treatment) for analysis. Cost data and operation statistics were collected from January 2018 to December 2018 through the operation financial reports and interviews with project managers, and community health workers. Common programmatic costs (indirect and overhead costs) were first calculated as total cost and were apportioned into each category. Unit cost for TB diagnosis with treatment per patient was recalculated from the direct costs of related diagnostic tool and medications according to the Morocco TB guideline, which is practiced in health centers in the Salé area of the Rabat-Sale-Kenitra region in Morocco.

**Table 1:** Parameter estimates for the cost-effectiveness analysis of the medication event monitoring systems for tuberculosis.

Parameters (Probability)	Base (Range)	Reference
TB treatment success rate in SoC	0.855 (0.7–0.86)	[13]
TB treatment success rate in MEMS	0.9897 (0.85–0.9948)^a^	[13, 20, 21]
Treatment failure or loss to f/u rate in SoC before 2 months	0.1298 (0.0604–0.14)	Used WHO Tuberculosis data files [17, 13, 14]
Treatment failure or loss to f/u rate in SoC after 2 months	0.1298 (0.0604–0.14)	Used WHO Tuberculosis data files [17, 13, 14]
Treatment failure or loss to f/u rate in MEMS before 2 months	0.0052 (0.0039–0.0065)	Used WHO Tuberculosis data files [17, 13, 14]
Treatment failure or loss to f/u rate in MEMS after 2 months	0.0052 (0.0039–0.0065)	Used WHO Tuberculosis data files [17, 13, 14]
Adherence rate for the initial 2 months in SoC	0.9924 (0.7443–1.0)	Used WHO Tuberculosis data files [17, 13, 14]
Adherence rate for the initial 2 months in MEMS	1 (0.75–1.00)	Used WHO Tuberculosis data files [17, 13, 14]
TB relapse after complete healing	0.054 (0.028–0.08)	[13, 22, 23]
Treatment failure or loss to f/u in the first MDR year	0.405 (0.392–0.417)	[24]
Mortality in the first MDR year	0.0405 (0.0393–0.0417)	[7, 9]
Treatment failure or loss to f/u in the second MDR year	0.405 (0.071–0.405)	[17, 24]
Mortality in the second MDR year	0.0405 (0.0393–0.0417)	[25, 26]
Mortality after MDR treatment success	0.001 (0.00097–0.00103)	[27]
Mortality after completely healing of MDR TB	0.001 (0.00097–0.00103)	[25, 27]
Mortality after loss to f/u or failure for MDR TB treatment	0.0405 (0.0393–0.0417)	[25, 27]
Relapse after MDR treatment	0.0925 (0.04–0.145)	[25–27]
TB re-treatment success rate	0.709 (0.636–0.7365)	[17, 25, 28]
TB re-treatment mortality rate	0.046 (0.0345–0.0575)	[25, 26, 28]
Completely healed after TB re-treatment	0.904 (0.838–0.904)	[17, 25, 28, 29]
Relapse rate after TB re-treatment	0.095 (0.028–0.095)	[23, 25, 28]
Mortality rate after loss to f/u or failure of TB re-treatment	0.078 (0.046–0.11)	Calculated from [23, 25, 29, 30]
MDR development after treatment failure or loss to f/u for retreatment	0.1685 (0.122–0.215)	[23, 25, 29, 31, 32]
**Mortality and Life Expectancy**		
TB or MDR TB (age-specific)	NA	WHO life table [27, 29, 31–33]
Completely healed (age-specific)	NA	WHO life table [27, 29, 31–33]
**Disutility**		
TB or MDR TB	0.331	[34]

^a^Base value was re-calculated excluding the non-evaluated cases

SoC = standard of care; MEMS = medication event monitoring system; MDR TB = multi-drug resistant tuberculosis; f/u = follow up; NA = non-applicable

**Table 2:** Unit costs for the treatment of tuberculosis per one patient in 2018 US$.

Cost components	Base (Range)	Reference
**Cost for the treatment of a new TB patient (6 months) in MEMS**	**398.70 (299.00–498.30)**	[13, 15]
**Managing cost for the initial 2 months**	**163.90**	
**Managing cost for the remaining 4 months**	**234.70**	
Cost for MEMS	219.40	Study
Installation and management cost for MEMS	99.80	Study
Staffing (Human Resources) for managing MEMS	77.80	Study
Overhead cost (office equipment and telephone use)	27.00	Study
Transportation for staffs’ visits	6.80	Study
Training and education, TB campaign (staffs and patients)	7.90	Study
Diagnosis and first-line treatment	132.10	Guidelines [15]
Staff time/salaries	47.20	Guidelines [15]
**Cost for the treatment of a new TB patient (6 months) in SoC**	**155.70 (116.70–194.50)**	[14, 15, 35]
**For the initial 2 months**	**84.80**	
**For the remaining 4 months**	**70.70**	
Overhead cost (Office equipment and telephone use)	4.80	Guidelines [15]
Transportation for staffs’ visits	3.40	Guidelines [15]
Diagnosis and first-line TB treatment	132.10	Guidelines [15]
Staff time/salaries	15.30	Guidelines [15]
**Recruiting TB patients who stopped TB treatment**	**5.10 (3.83–6.38)**	[15]
**Cost for TB retreatment (not MDR, 8 months)**	**762.20 (571.60–952.80)**	[15, 32]
Diagnosis and treatment	676.50	Guidelines [15]
Staff time/salaries	85.70	Guidelines [15]
**Cost for MDR Treatment for 24 months** ^a^	**4437.30 (3327.90–4159.90)**	[15, 24]
**For the first year**	**2841.60**	
**For the second year**	**1595.60**	
Diagnosis and treatment per MDR TB	4138.40	Guidelines [15]
Staff time/salaries	298.80	Guidelines [15]

^a^This cost was calculated for the outpatient treatment of an MDR-TB patient

TB = tuberculosis; MEMS = medication event monitoring system; SoC = standard of care; MDR TB = multi-drug resistant tuberculosis

Human resource costs for each activity component were calculated based on the approximate time engaged in each activity during the operations, assessed using a workload survey. For the costs of goods, equipment, and services, we divided direct costs into capital and recurrent costs. Capital costs were annualized over its expected remaining lifetime, and discounted at a 3% annual rate. Costs were collected as Moroccan Dirhams (MAD), and were converted as 2018 US dollars ($US) using the standard inflation adjustment method, at the rate of 9.116 Moroccan Dirhams (MAD) per US$1, based on the average UN exchange reported for 2018.

### Model outcomes

The primary outcome of analysis was the incremental cost-effectiveness ratio (ICER), measured in units of 2018 US$ per disability adjusted life-year (DALY) averted. For DALY calculation, the life table of Morocco and the raw data of the WHO reports were adopted [17]. We also estimated the total number of treatment-related acquired MDR-TB and mortality. Future DALYs and costs were discounted at 3% per year. We performed one- and two-way deterministic sensitivity analyses for all model parameters using a range of parameter values based on the literature review and the project data when available, or ±25% of baseline values. For the Probabilistic Sensitivity Analysis (PSA), all model parameter values were randomly sample over 1,000 Monte Carlo simulations based on pre-specified distributions of each parameter to generate 95% uncertainty ranges.

### Ethical approval and consent to participate

The consent for this study was waved, as it was based on a simulated cohort of patients. This study was approved by the Institution Review Board (IRB) of Mohammed V University (IRB number: Dossier number 48/16)

## Results

### Treatment-related costs

Table 2 represents the unit costs and total costs under the MEMS and SoC. The length of each treatments are 6 month for initial treatment, 8months for retreatment, and 20 months for MDR-TB treatment. The average total per-patient health system costs for treating a new TB patient using the MEMS and SoC were $398.70 and $155.70, respectively. For a new TB patient using the MEMS, the installation and management cost of MEMS was $99.80, while the cost for human resources managing MEMS and overhead costs was $77.80 and $27.00 respectively. Among $77.80 for human resources, the additional cost for phone calls and visits done by staffs, related with feedback from the device, were $3.80 and $6.30, respectively. The cost of staff time and salaries for patient care in health clinics was $47.20. For both strategies, the cost of diagnosis and treatment was $132.10. The diagnosis and treatment costs for nine months of re-treatment for drug-susceptible TB and 24 months of MDR-TB treatment were $762.20 and $4,437.30, respectively.

### Effectiveness and cost-effectiveness

Under the SoC strategy, 0.48 drug-susceptible TB cases and 0.03 MDR-TB cases / per patient (first year) would occur over the 5-year time horizon (Table 3). The developed total TB cases and the completely healed cases from TB including MDR would be 0.53 cases and 0.41 cases / per patient, respectively. However, under the MEMS strategy, 0.31 drug-susceptible TB and 0.02 MDR-TB cases / per patient (first year) would occur. The developed total TB cases and the completely healed cases from TB including MDR would be 0.34 cases and 0.62 cases / per patient, respectively. Therefore, MEMS strategy would reduce the number of drug-susceptible TB cases by 0.17 and MDR-TB cases by 0.01 per patient over 5 years. The expected total costs and DALYs lost per patient over 5 years under the SoC strategy were $619 and 0.52 DALYs, respectively. Under the MEMS strategy, the expected total costs and DALYs lost per patient over 5 years were $745 and 0.23 DALYs, respectively. The ICER was $434/DALY averted by MEMS relative to SoC. In probabilistic sensitivity analysis, compared with SoC, MEMS reduced DALYs by 0.20 (95% uncertainty range, 0.08–0.32) with an increased cost of US$125 (95% uncertainty range, 93–158) (S1). In addition, the probabilities of each strategy were presented in the acceptability curves over a range of WTP (0–100,000 US$/DALY) (S2). The MEMS is accepted as cost-effective in 100% of instances at a WTP threshold ≥ 3,000 US$/DALY.

**Table 3:** Expected total costs, DALY, and incremental cost-effectiveness per patient over 5 years.

Strategy	Cost, US$	Number of drug-susceptible TB cases	Number of MDR-TB cases	Deaths	DALYs	Incremental cost/DALY averted US$
SoC	619	0.48	0.03	0.06	0.52	-
MEMS	745	0.31	0.02	0.04	0.23	-
Incremental	125	-0.17	-0.01	-0.02	-0.29	434

DALY = disability adjusted life-year; SoC = standard of care; MEMS = Medication event monitoring system

### Sensitivity analyses

**Fig 3:**
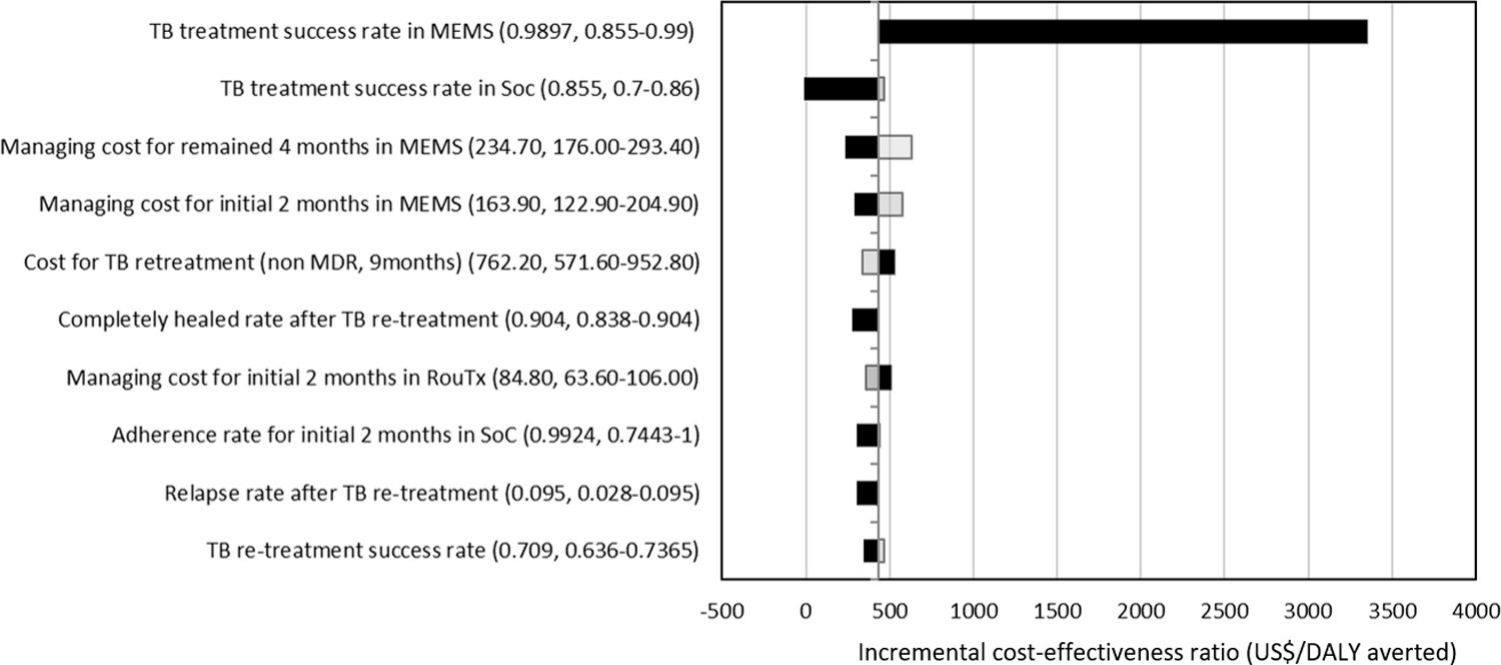
One-way sensitivity tornado diagram comparing routine treatment and MEMS. Bars represent the incremental cost-effectiveness ratio under the low (black) and high (light gray line) bounds associated with each parameter. TB = tuberculosis; MEMS = medication event monitoring system; SoC = standard of care; MDR = multi-drug resistant tuberculosis; DALY disability adjusted life-year.

**Fig 4:**
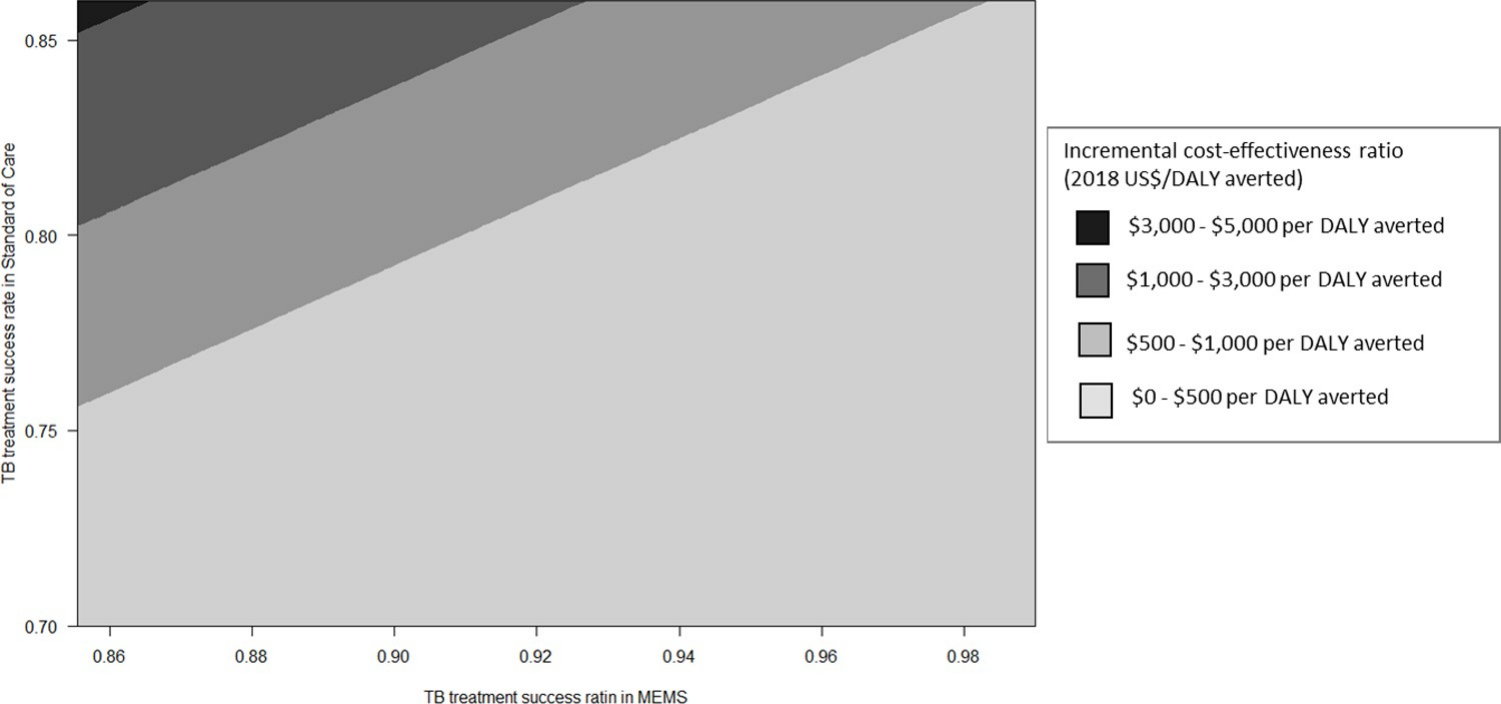
Incremental cost-effectiveness of TB treatment according to the treatment success rate of MEMS versus routine treatment. Each shaped region corresponds to a range of incremental cost-effectiveness ratios (in 2018 US$/DALY averted) for the TB treatment success rate in MEMS relative to that in routine treatment. The x-axis denotes the different probabilities of TB treatment success rates in MEMS, and the y-axis for routine treatment. The different line border denotes the border of willingness to pay threshold, and the lighter area favors TB treatment with MEMS at each willingness to pay threshold. MEMS = medication event monitoring system; DALY = disability-adjusted life-year.

In tornado diagram of ten influential factors identified in one-way sensitivity analysis (Fig 3), the ICER of TB management was most sensitive to the TB treatment success rate in MEMS and TB treatment success rate in SoC, followed by managing costs of MEMS during continuation and intensive phases. When we assumed a lower margin of TB treatment success rate in MEMS as 85%, the ICER was $3,350/DALY averted at this point. In two-way sensitivity analysis (Fig 4), at a WTP threshold of $500/DALY and $1,000/DALY averted, 70.1% and 91.1% favored TB treatment with the MEMS, respectively. In probabilistic sensitivity analysis with 1,000 Monte Carlo simulations, at a WTP threshold of $3,000 (the per-capita gross national income of Morocco), MEMS was cost-effective compared with SoC in the nearly 100% of instances (S1 Fig).

## Discussion

We found that MEMS intervention in Morocco would cost $434/DALY averted for managing drug-susceptible TB patients with AFB smear-positive sputum over 5 years of time horizon, compared to SoC. The MEMS strategy would reduce the number of drug-susceptible TB by 0.17 and MDR-TB by 0.01 per patient over 5 years. The result was most sensitive to the treatment success rate of drug-susceptible TB using the MEMS strategy. In light of the GDP of Morocco ($3,000), our study results support that MEMS is considered cost-effective in Morocco.

The strength of our study is that we included micro-costing and measured the detailed costs of implementing MEMS and the standard care of drug-susceptible TB and MDR-TB, including staff salaries, follow-up visits, and costs for monitoring tests and drugs. The estimated total cost of MDR treatment in our study was lower than that reported from other studies [8]. Potential reasons are that we did not include any costs related to in-patient treatment, and the costs for treatment and human resources in Morocco are relatively lower compared to other countries [8]. However, the increased feedback such as visits and phone calls by community health workers, responding to the decreased adherence rate in the remained 4 month of TB treatment, could contribute the higher cost for MEMS as the main influential factor to ICER change, apart from the higher cost of examination and treatment.

The key factor driving a relatively low ICER for MEMS was the higher success rate of TB treatment with MEMS. There has been limited evidence of MEMS effectiveness on percentage of cure and completion as the indicators for treatment success [18]. In a small study in South Africa, the TB cure rate using medication monitors was reported to be 75–100% [7]. In Uganda, the treatment success rate using e-adherence software and mobile phone reminders was 80.6% and 96.7%, respectively [19]. We varied the TB treatment success rate in a reasonable wide range from 0.85 to 0.99 in the sensitivity analysis and found that almost 100% favored the MEMS strategy at the threshold of $3,000/DALY averted.

In addition to the TB treatment success rate, the ICER was also sensitive to the managing costs of MEMS during continuation and intensive phases. In another study in Brazil, the unit cost of TB medication monitor was $423, which was slightly higher than the unit cost per person ($398.70) in our study [8]. More studies are needed to determine the cost-effectiveness of MEMS in settings with different TB burdens, feasibilities for digital health, and costs for TB management in order to settle the program. However, Morocco Ministry of Health is on scaling up MEMS program through the local TB association, integrated with sustainable community, and this MEMS strategy could be transferred to other Francophonie countries in cooperation with technology upgrades.

Our study has several limitations. First, MEMS only allows to monitor opening of the pillboxes electronically. Therefore, drug adherence is not guaranteed without virtual confirmation of the medication counts. Second, costs for MDR-TB were assumed based on outpatient clinics only and did not include any costs for in-patient treatment. Third, we did not include bedaquline and delamanid as MDR-TB new drugs. Fourth, actual data were used only for drug-susceptible TB, and we used the data from literature reviews to estimate the costs related to recurring TB and MDR-TB management. Lastly, because the original KOICA study that provided epidemiologic parameter for this analysis had been a non-randomized study, the input data for this cost-effective analysis could not have been fully adjusted, reflecting the difference of patients’ characteristics and behavior between the enrolled MEMS and SoC group. Therefore, considering much higher medical costs and hindrance to actual data gathering for MDR-TB as well as retreated DS-TB, a deeper investigation based on more reliable data is needed.

## Conclusions

Our study demonstrated the cost-effectiveness of MEMS among infectious active TB patients in Morocco. To inform scale-up of digital health intervention as part of TB control strategy, further cost-effectiveness studies are needed under different national tuberculosis programs and TB burdens.

## Supporting information

S1 Fig Probabilistic Sensitivity Analysis (PSA) with 1,000 Monte Carlo simulations with a WTP threshold of $3,000 (the per-capita gross national income of Morocco). (TIF)

S2 Fig The acceptability curves over a range of WTP (0–100,000 US$/DALY). (TIF)
