# Supplementary figures and images for: Cost-effectiveness of a medication event monitoring system for tuberculosis management in Morocco
Source: PLoS One. 2022 Apr 19;17(4):e0267292. doi: 10.1371/journal.pone.0267292 (PMC9017941; doi:10.1371/journal.pone.0267292)

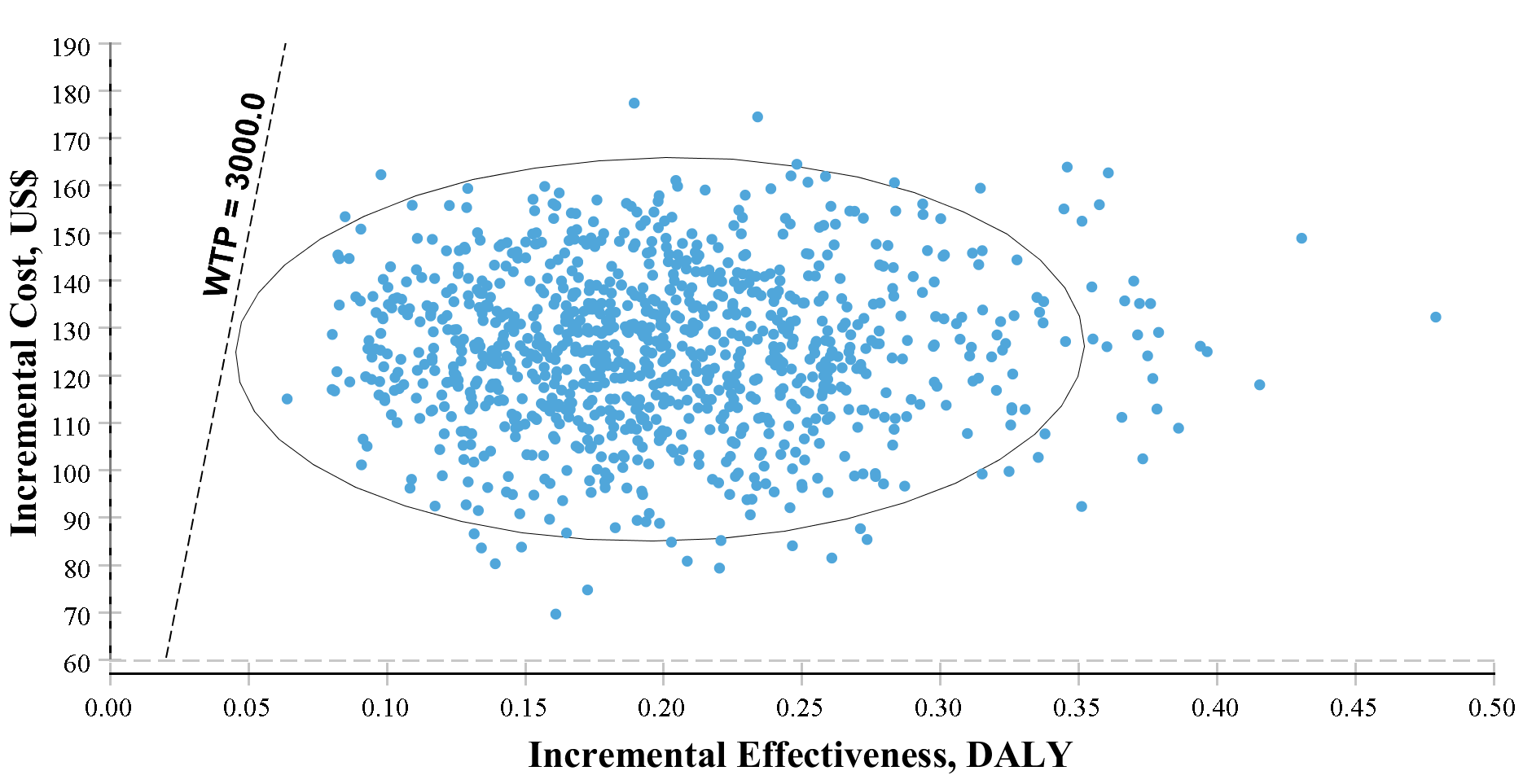

Supplement: S1 Fig — (TIF) [file pone.0267292.s001.tif]

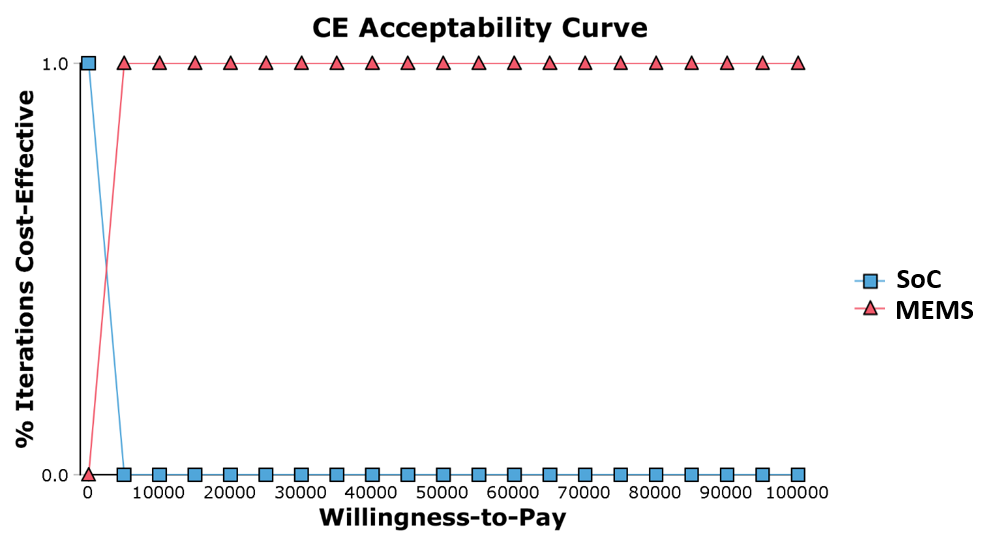

Supplement: S2 Fig — (TIF) [file pone.0267292.s002.tif]
